# The complete chloroplast genome sequence of *Cypripedium tibeticum* King Ex Rolfe (Orchidaceae)

**DOI:** 10.1080/23802359.2019.1698357

**Published:** 2019-12-12

**Authors:** Jianfang Li, Bei Xu, Qian Yang, Zhan-Lin Liu

**Affiliations:** Key Laboratory of Resource Biology and Biotechnology in Western China (Ministry of Education), College of Life Sciences, Northwest University, Xi’an, Shaanxi, China

**Keywords:** *Cypripedium tibeticum*, Orchidaceae, plastome, phylogeny

## Abstract

In this study, we determined the complete chloroplast genome of *Cypripedium tibeticum*, an endangered species in China. The plastome is 159,223 bp in length, with a large single-copy region (LSC) of 86,537 bp, a small single-copy region (SSC) of 17,552 bp, and a pair of inverted repeat regions (IRs) of 27,567 bp each. It contains 133 genes, including 87 protein-coding genes, 38 tRNA genes, and 8 rRNA genes. The overall GC content was 36.9%, while the corresponding values in the LSC, SSC, and IR regions are 34.6, 30.4, and 42.6%, respectively. The phylogenetic analysis showed that *C. tibeticum* was closely related to its congeners and the classification of five subfamilies of Orchidaceae was also highly supported.

The Orchidaceae is well known for the economic, ecological, cultural values and all species are classified as endangered plants for the overexploitation of wild resources. The phylogenetic relationships among species and/or groups are often changeable, mainly due to the enormous number in the family. Recent molecular studies strongly supported that Orchidaceae could be classified into five subfamilies (Givnish et al. 2015). *Cypripedium*, belonging to the subfamily of Cypripedioideae, contains about 50 species with widely vegetative and floral variations. The infrageneric classification in the genus remains incompletely identified and phylogenomic works are necessarily needed in the future (Li et al. 2011). *Cypripedium tibeticum* King ex Rolfe is mainly distributed in western China, Bhutan, and Sikkim. Its roots are commonly used as medicine for the treatment of rheumatism and edema (Teoh 2016). In this study, we determined the complete chloroplast genome of *C. tibeticum* to provide genomic information for conservation management of the species and phylogenetic research in Orchidaceae.

Total genomic DNA was extracted from fresh leaves of an individual of *C. tibeticum* collected in Qinling Mountains, China (N33.52°, E108.54°). The voucher (201408734) was deposited at the Evolutionary Botany Laboratory (EBL), Northwest University. Data processing follows the previous studies (Li et al. 2019; Peng et al. 2019), including genome sequencing, reads trimming/assembling and gene annotation. The plastome was annotated with *Cypripedium japonicum* (NC 027227) as reference and has been deposited into GenBank with the accession number of MN561380.

The whole chloroplast genome of *C. tibeticum* is 159,223 bp, including a large single-copy region (LSC) of 86,537 bp, a small single-copy region (SSC) of 17,552 bp, and a pair of inverted repeat regions (IRs) of 27,567 bp each. A total of 133 genes were detected, including 87 protein-coding genes, 38 tRNA genes, and 8 rRNA genes. Eighteen genes are duplicated in the IRs, containing six protein-coding genes (*rps*19, *rpl*2, *rpl*23, *ycf*2, *ndh*B, and *rps*7), eight tRNA genes (*trn*H-GUG, *trn*I-CAU, *trn*L-CAA, *trn*V-GAC, *trn*I-GAU, *trn*A-UGC, *trn*R-ACG, and *trn*N-GUU), and four rRNA genes (*rrn*16, *rrn*23, *rrn*4.5, *rrn*5). Among the annotated genes, 15 contain a single intron, including 9 protein-coding genes (*rps*16, *atp*F, *rpo*C1, *pet*B, *pet*D, *rpl*16, *rpl*2, *ndh*B, and *ndh*A), and 6 tRNAs (*trn*K-UUU, *trn*G-UCC, *trn*L-UAA, *trn*V-UAC, *trn*I-GAU, and *trn*A-UGC), and 3 genes (*rps*12, *clp*P, and *ycf*3) harbored two introns. The overall GC content was 36.9%, while the corresponding values in the LSC, SSC, and IR regions are 34.6, 30.4, and 42.6%, respectively.

For the phylogenetic analysis, 24 plastomes from representative species in Orchidaceae were used to conduct the maximum-likelihood tree following the previous study (Peng et al. 2019) with *Iris sanguinea* (NC_029227) and *Narcissus poeticus* (NC_039825) as outgroup. Our result showed that *C. tibeticum* was a sister to *C. japonicum* and the genus was clustered with other genera of Cypripedioideae (Figure 1). The monophyly and relationships of five subfamilies of Orchidaceae were also well supported by our data.

**Figure 1.**
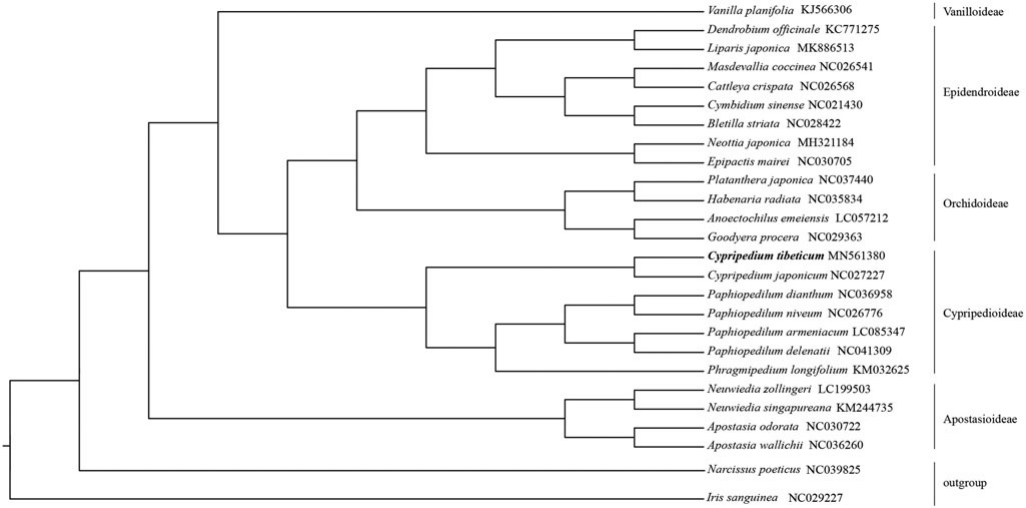
The phylogenetic tree of representative species in Orchidaceae constructed by maximum-likelihood method using the whole plastome sequences. All branches were 100% supported with 1000 bootstrap replicates.
